# An asymptotic higher-order theory for rectangular beams

**DOI:** 10.1098/rspa.2018.0001

**Published:** 2018-06-13

**Authors:** E. Nolde, A. V. Pichugin, J. Kaplunov

**Affiliations:** 1 Department of Mathematics, CEDPS, Brunel University London, Uxbridge UB8 3PH, UK; 2 School of Computing and Mathematics, Keele University, Keele, Staffordshire ST5 5BG, UK

**Keywords:** asymptotics, long waves, low frequency, beam theory, Timoshenko beam theory, shear correction factor

## Abstract

A direct asymptotic integration of the full three-dimensional problem of elasticity is employed to derive a consistent governing equation for a beam with the rectangular cross section. The governing equation is consistent in the sense that it has the same long-wave low-frequency behaviour as the exact solution of the original three-dimensional problem. Performance of the new beam equation is illustrated by comparing its predictions against the results of direct finite-element computations. Limiting behaviours for beams with large (and small) aspect ratios, which can be established using classical plate theories, are recovered from the new governing equation to illustrate its consistency and also to illustrate the importance of using plate theories with the correctly refined boundary conditions. The implications for the correct choice of the shear correction factor in Timoshenko's beam theory are also discussed.

## 1. Introduction

The classical Euler–Bernoulli equation can be interpreted in two ways. On the one hand, it can be understood as an approximate equation that broadly reproduces behaviour of thin beams at sufficiently low frequencies. This interpretation is valid and, in fact, this is how the Euler–Bernoulli equation is most commonly introduced, typically justified by *ad hoc* strength of materials style arguments. On the other hand, another, more mathematically precise definition is also possible. One can start with a full three-dimensional boundary value problem for a prism composed of an elastic material and consider a *long-wave low-frequency* asymptotic expansion under the assumptions that frequencies of interest are low and characteristic dimensions of the cross section are negligible in relation to the wavelength of interest. It can be shown that the Euler–Bernoulli equation recovers the leading-order term of this expansion. In this sense, the classical theory is precisely defined and fully mathematically consistent. This also suggests that further terms in the same asymptotic expansion can be considered to, hopefully, construct more precise, yet still asymptotically consistent approximations to the full three-dimensional solution. This is precisely the topic of our discussion.

It may seem surprising that higher-order equations for a beam can still warrant an investigation. After all, more geometrically complex objects, such as plates and shells, have well-developed higher-order theories (e.g. [1–4]). The explanation is that cross-sectional problems for both plates and shells are always one-dimensional and typically possess simple analytical solutions, whereas cross-sectional problems for beams are necessarily two-dimensional, which makes their solution substantially harder. It is also worth noting that deriving new higher-order equations is not the goal in itself; instead, one can use them to construct higher-order asymptotic solutions for much harder boundary value problems, e.g. vibration of elliptic plates [5] or scattering of acoustic waves by an immersed shell [6] or solution of initial-value problems [7].

The most popular refined beam equation was obtained by Timoshenko [8] (see recent review [9]). It is derived using several *ad hoc* assumptions and, as a result, does not generally predict the correct higher-order asymptotic behaviour of the beam. It features two vibration modes, a lower (fundamental) mode that describes bending, as well as a higher mode that bears only superficial similarity to the true three-dimensional solution, see [10]. Nevertheless, predictions by Timoshenko's governing equation can be made partly consistent because the equation features an imprecisely defined constant *κ*, usually called the shear correction factor. It turns out that *κ* can be chosen in such way that the fundamental mode of Timoshenko's theory matches the appropriate expansion of the corresponding exact three-dimensional solution, see [11].

Berdichevskii & Kvashnina [12] were apparently the first who attempted to construct a more consistent equivalent of Timoshenko's equation using the variational asymptotic method. Unfortunately, when specialized to the case of rectangular beams, their equation appears to contain an erroneous coefficient, this will be further discussed in §7. The methodology of Berdichevskii and Kvashnina was generalized in [13–15], focusing mainly on derivations of the approximations of the potential energy.

The goal of this paper is to present the derivation of a truly asymptotically consistent higher-order theory for rectangular beams. Our derivation is based upon the direct asymptotic integration of the exact three-dimensional problem of elasticity. This method has been originally developed by Goldenveizer, see e.g. [16], as well as more recent paper [3]. Such an approach allows one to construct internally consistent higher-order approximations for the stress and strain fields across the cross section. This makes our approach largely similar to [17], in which a more general problem for a beam composed of an anisotropic elastic material has been considered. The main difference is that the coefficients of beam equation in [17] were obtained by solving a cross section problem numerically, whereas we construct a governing equation with fully explicit analytical expressions for all coefficients.

The restriction to the rectangular cross section enables us to obtain a fully analytical description of the simplest non-trivial configuration of the beam, the classical benchmark problem that we believe is still not resolved in the literature conclusively. We validate new beam equation by showing that the dispersion curves by our theory faithfully reproduce the long-wave low-frequency behaviour of the exact dispersion curves computed using the SAFE method for several aspect ratios of the beam. The choice of rectangular cross section also allows us to provide an analytical validation of the results. Specifically, motion of rectangular beams with large or small aspect ratios can be described by the classical plate theories: the out-of-plane bending mode can be reproduced as symmetric fundamental mode of a Kirchhoff plate strip and the in-plane bending mode can be reproduced by studying antisymmetric fundamental mode of a plate strip in the state of plane stress. The Kirchhoff plate strip limit is particularly interesting, because we show that the use of refined boundary conditions for the plate theory by Kolos [18] allows to reproduce the correct asymptotic behaviour of even a moderately thick flat beam.

It is instructive to compare the predictions by our equation to the response of Timoshenko's beam equation. We do this by matching the relevant dispersion curves; this allows us to propose a new expression for the shear correction factor *κ*. In his papers, Timoshenko used constant (cross-section-independent) values of *κ*, see [8,19]; the most commonly cited reference [20] provides values of *κ* for a number of canonical cross sections that also depend on the Poisson ratio *ν*. However, the coefficients given in [20] are known to be asymptotically inconsistent, at least for circular and rectangular cross sections, see [11]. A particular expression of *κ*, first obtained in [21], was independently confirmed in [22–25]. The expression for value of *κ* for rectangular beams obtained in this paper confirms the consistency of the coefficient proposed in [21]. In addition, we demonstrate that a number of other definitions of shear correction factor given in the recent literature do not lead to a consistent form of Timoshenko's equation.

## 2. Governing equations

Consider an infinite rectangular beam composed of a homogeneous isotropic elastic material. Motions of isotropic media are governed by the equations of three-dimensional elasticity

2.1 $$\tau_{ij,j}=\rho {\ddot{u}}_{i},\;\tau_{ij}=\frac{E}{1+\nu }\left(\frac{\nu \epsilon_{kk}\delta_{ij}}{1-2\nu }+\epsilon_{ij}\right),\;\epsilon_{ij}=\frac{1}{2}(u_{i,j}+u_{j,i}),$$

where $\tau_{ij}=\tau_{ij}(x,t)$ is the stress tensor, $u_{i}=u_{i}(x,t)$ the displacement vector, *ρ* the mass density per unit volume, *E* the Young modulus and *ν* the Poisson ratio. Comma subscripts in (2.1) indicate differentiation with respect to implied spatial coordinates; henceforth we will also use comma subscripts to denote differentiation with respect to explicitly shown non-dimensional spatial coordinates and time. Also assumed specifically in (2.1) are the summation over repeated suffices and overdots denoting differentiation with respect to time *t*.

**Figure 1.**
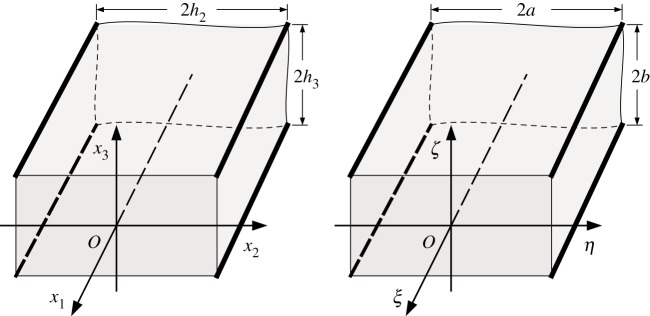
The dimensional and non-dimensional coordinate systems for the considered problem.

We place the origin of the Cartesian coordinate system at the symmetry axis of the beam and direct axis *Ox*_1_ along the symmetry axis. The beam cross section is the rectangle {(*x*_2_,*x*_3_):−*h*_2_≤*x*_2_≤*h*_2_,−*h*_3_≤*x*_3_≤*h*_3_} (figure 1). The boundary conditions on the free faces of the beam may then be written as

2.2 $$\begin{array}{rl} \tau_{2i} & \;=0\;(i=1,2,3)\;\text{at}\;x_{2}=\pm h_{2}\\ \text{and}\;\;\tau_{3i} & \;=0\;(i=1,2,3)\;\text{at}\;x_{3}=\pm h_{3}. \end{array}$$

Our primary interest is with the propagation of long waves, i.e. waves whose wavelength *l* is much greater than a characteristic dimension of the cross section *h*.^1^ This suggests introducing a natural small parameter *ε*=*h*/*l* and re-scaling the problem in terms of the following non-dimensional coordinates:

2.3 $$x_{1}=l\xi ,\;x_{2}=\varepsilon l\eta ,\;x_{3}=\varepsilon l\zeta ,\;t=\frac{\varepsilon^{-1}l\tau }{\sqrt{E/\rho }}.$$

An additional assumption that the beam bends along the axis *Ox*_3_ prompts an appropriate re-scaling for the displacement and stress components:

2.4 $$\begin{array}{rl} u_{1} & \;=\varepsilon lu,\;u_{2}=\varepsilon^{2}lv,\;u_{3}=lw,\\ \tau_{11} & \;=\varepsilon E\sigma_{11},\;\tau_{33}=\varepsilon^{3}E\sigma_{33},\\ \tau_{1i} & \;=\varepsilon^{2}E\sigma_{1i},\;\tau_{2i}=\varepsilon^{3}E\sigma_{2i}\;(i=2,3). \end{array}$$

The introduction of scalings (2.4) can be partly motivated by referring to the classical Euler–Bernoulli theory and also by the asymptotic analysis of the exact dispersion relations for similar problems (e.g. for the bending of a circular beam). Here, for the sake of brevity, we introduce the ansatz constructively and prove its consistency *a posteriori*, by observing that the proposed form of the asymptotic series satisfies appropriate equations and boundary conditions at all orders.

In terms of the newly introduced non-dimensional quantities, we can re-write governing equations (2.1) in the following form:

2.5 $$\begin{array}{rl} \sigma_{11,\xi }+\sigma_{12,\eta }+\sigma_{13,\zeta } & \;=\varepsilon^{2}u_{,\tau \tau },\\ \sigma_{21,\xi }+\sigma_{22,\eta }+\sigma_{23,\zeta } & \;=\varepsilon^{2}v_{,\tau \tau },\\ \sigma_{31,\xi }+\sigma_{32,\eta }+\sigma_{33,\zeta } & \;=w_{,\tau \tau }, \end{array}$$

and

2.6 $$\begin{array}{rl} \varepsilon^{2}(1+\nu )(1-2\nu )\sigma_{11} & \;=\varepsilon^{2}(1-\nu )u_{,\xi }+\varepsilon^{2}\nu v_{,\eta }+\nu w_{,\zeta },\\ \varepsilon^{4}(1+\nu )(1-2\nu )\sigma_{22} & \;=\varepsilon^{2}\nu u_{,\xi }+\varepsilon^{2}(1-\nu )v_{,\eta }+\nu w_{,\zeta },\\ \varepsilon^{4}(1+\nu )(1-2\nu )\sigma_{33} & \;=\varepsilon^{2}\nu u_{,\xi }+\varepsilon^{2}\nu v_{,\eta }+(1-\nu )w_{,\zeta },\\ 2\varepsilon^{2}(1+\nu )\sigma_{12} & \;=u_{,\eta }+\varepsilon^{2}v_{,\xi },\\ 2\varepsilon^{2}(1+\nu )\sigma_{13} & \;=u_{,\zeta }+w_{,\xi },\\ \text{and}\;\;2\varepsilon^{4}(1+\nu )\sigma_{23} & \;=\varepsilon^{2}v_{,\zeta }+w_{,\eta }. \end{array}$$

Boundary conditions (2.2) can also be appropriately re-formulated as

2.7 $$\begin{array}{rl} \sigma_{2i} & \;=0\;(i=1,2,3)\;\text{at}\;\eta =\pm a\\ \text{and}\;\;\sigma_{3i} & \;=0\;(i=1,2,3)\;\text{at}\;\zeta =\pm b, \end{array}$$

where *a*=*h*_2_/*h* and *b*=*h*_3_/*h*.

The solution *f*={*u*,*v*,*w*,*σ*_*ij*_} of the boundary value problem (2.5)–(2.7) is now sought in form of the following asymptotic ansatz:

2.8 $$f(\xi ,\eta ,\zeta ,\tau )=f^{\left(0\right)}+\varepsilon^{2}f^{\left(2\right)}+\varepsilon^{4}f^{\left(4\right)}+\cdots ,$$

where *f*^(*k*)^=*f*^(*k*)^(*ξ*,*η*,*ζ*,*τ*) and

2.9 $$f^{\left(k\right)}(\xi ,\eta ,\zeta ,\tau )=\sum_{m}{\tilde{f}}_{m}^{\left(k\right)}(\xi ,\tau )F_{m}^{\left(k\right)}(\eta ,\zeta ).$$

## 3. The leading-order theory

The leading-order displacements are governed by the leading-order terms of governing equations (2.6). The relevant equations can be obtained by substituting expansion (2.8) into equations (2.6) and neglecting all terms that are of *O*(*ε*^2^) (and smaller), which yields

3.1 $$w_{,\zeta }^{\left(0\right)}=0,\;u_{,\eta }^{\left(0\right)}=0,\;u_{,\zeta }^{\left(0\right)}+w_{,\xi }^{\left(0\right)}=0,\;w_{,\eta }^{\left(0\right)}=0.$$

The appropriate solution is given by

3.2 $$w^{\left(0\right)}={\tilde{w}}_{0},\;u^{\left(0\right)}=-{\tilde{w}}_{0}^{\prime }\zeta ,$$

where ${\tilde{w}}_{0}={\tilde{w}}_{0}(\xi ,\tau )$ and a dash denotes differentiation with respect to *ξ*.

The governing equations for the next-order displacements and remaining leading-order quantities have the form

3.3 $$\begin{array}{rl} \sigma_{11,\xi }^{\left(0\right)}+\sigma_{12,\eta }^{\left(0\right)}+\sigma_{13,\zeta }^{\left(0\right)} & \;=0, \end{array}$$

3.4 $$\begin{array}{rl} \sigma_{21,\xi }^{\left(0\right)}+\sigma_{22,\eta }^{\left(0\right)}+\sigma_{23,\zeta }^{\left(0\right)} & \;=0, \end{array}$$

3.5 $$\begin{array}{rl} \sigma_{31,\xi }^{\left(0\right)}+\sigma_{32,\eta }^{\left(0\right)}+\sigma_{33,\zeta }^{\left(0\right)} & \;={\tilde{w}}_{,\tau \tau }, \end{array}$$

and

3.6 $$\begin{array}{rl} (1+\nu )(1-2\nu )\sigma_{11}^{\left(0\right)} & \;=(1-\nu )u_{,\xi }^{\left(0\right)}+\nu v_{,\eta }^{\left(0\right)}+\nu w_{,\zeta }^{\left(2\right)}, \end{array}$$

3.7 $$\begin{array}{rl} 0 & \;=\nu u_{,\xi }^{\left(0\right)}+(1-\nu )v_{,\eta }^{\left(0\right)}+\nu w_{,\zeta }^{\left(2\right)}, \end{array}$$

3.8 $$\begin{array}{rl} 0 & \;=\nu u_{,\xi }^{\left(0\right)}+\nu v_{,\eta }^{\left(0\right)}+(1-\nu )w_{,\zeta }^{\left(2\right)}, \end{array}$$

3.9 $$\begin{array}{rl} 2(1+\nu )\sigma_{12}^{\left(0\right)} & \;=u_{,\eta }^{\left(2\right)}+v_{,\xi }^{\left(0\right)}, \end{array}$$

3.10 $$\begin{array}{rl} 2(1+\nu )\sigma_{13}^{\left(0\right)} & \;=u_{,\zeta }^{\left(2\right)}+w_{,\xi }^{\left(2\right)}, \end{array}$$

3.11 $$\begin{array}{rl} 0 & \;=v_{,\zeta }^{\left(0\right)}+w_{,\eta }^{\left(2\right)}. \end{array}$$

Using the leading-order solution (3.2) and taking into account the (anti-) symmetry of the problem allow one to integrate governing equations (3.7), (3.8) and (3.11) with the following result:

3.12 $$v^{\left(0\right)}=\nu {\tilde{w}}_{0}^{\prime \prime }\eta \zeta ,\;w^{\left(2\right)}=\frac{1}{2}\nu {\tilde{w}}_{0}^{\prime \prime }(\zeta^{2}-\eta^{2})+{\tilde{w}}_{2},$$

where ${\tilde{w}}_{2}={\tilde{w}}_{2}(\xi ,\tau )$. Governing equation (3.6) then gives

3.13 $$\sigma_{11}^{\left(0\right)}=-{\tilde{w}}_{0}^{\prime \prime }\zeta .$$

Function ${\tilde{w}}_{0}$ underlying the leading-order solution still has not been specified. It can be determined by multiplying equation (3.3) by *ζ* and integrating over the cross section represented by rectangular region *R*, where

3.14 R={(η,ζ)|−a≤η≤a,−b≤ζ≤b}.

In the view of the boundary conditions at *η*=±*a* and *ζ*=±*b*, this yields

3.15 $$\iint_{R}\frac{\partial \sigma_{13}^{\left(0\right)}}{\partial \xi }\;d\eta \;d\zeta =-\frac{4}{3}ab^{3}{\tilde{w}}_{0}^{\prime \prime \prime }.$$

Similar integration of (3.5) over *R* gives then the equation for ${\tilde{w}}_{0}$

3.16 $$\frac{1}{3}b^{2}\frac{\partial^{4}{\tilde{w}}_{0}}{\partial \xi^{4}}+\frac{\partial^{2}{\tilde{w}}_{0}}{\partial \tau^{2}}=0,$$

which can be recognized as the classical Euler–Bernoulli equation for a rectangular beam. Indeed, given that ${\tilde{w}}_{0}∼w=u_{3}/l$, we can formulate the dimensional equivalent of equation (3.16) as

3.17 $$\frac{Eh_{3}^{2}}{3\rho }\frac{\partial^{4}u_{3}}{\partial x_{1}^{4}}+\frac{\partial^{2}u_{3}}{\partial t^{2}}=0.$$

Keeping in mind that the second moment for rectangular cross section *I*_*x*_2__=2*h*_2_(2*h*_3_)^3^/12 and the cross-sectional area *A*=4*h*_2_*h*_3_, the coefficient in front of the fourth-order term can now be recognized as the usual constant *EI*_*x*_2__/*ρA*.

To obtain an expression for *u*^(2)^, we first express $\sigma_{12}^{\left(0\right)}$ and $\sigma_{13}^{\left(0\right)}$ from equations (3.9) and (3.10), and then substitute the resulting expressions into (3.3) and boundary conditions $\sigma_{12}^{\left(0\right)}{|}_{\eta =\pm a}=0$ and $\sigma_{13}^{\left(0\right)}{|}_{\zeta =\pm b}=0$. As a result of these manipulations, we are led to the following boundary value problem:

3.18 $$\begin{array}{c} u_{,\eta \eta }^{\left(2\right)}+u_{,\zeta \zeta }^{\left(2\right)}=2{\tilde{w}}_{0}^{\prime \prime \prime }\zeta ,\\ u_{,\eta }^{\left(2\right)}{|}_{\eta =\pm a}=\mp \nu {\tilde{w}}_{0}^{\prime \prime \prime }a\zeta ,\;u_{,\zeta }^{\left(2\right)}{|}_{\zeta =\pm b}=\frac{1}{2}\nu {\tilde{w}}_{0}^{\prime \prime \prime }(\eta^{2}-b^{2})-{\tilde{w}}_{2}^{\prime }. \end{array}$$

We are seeking the solution of problem (3.18) in the form

3.19 $$u^{\left(2\right)}={\tilde{w}}_{0}^{\prime \prime \prime }U^{\left(2\right)}(\eta ,\zeta )-{\tilde{w}}_{2}^{\prime }\zeta .$$

It is easy to verify that if one takes

3.20 $$U^{\left(2\right)}=\frac{1}{6}(2-\nu )\zeta^{3}-b^{2}\zeta +\frac{1}{2}\nu \zeta \eta^{2}+\Psi (\eta ,\zeta ),$$

then the problem (3.18) is reduced to the boundary value problem

3.21 $$\begin{array}{c} \Psi_{,\eta \eta }+\Psi_{,\zeta \zeta }=0,\\ \Psi_{,\eta }{|}_{\eta =\pm a}=\mp 2\nu a\zeta ,\;\Psi_{,\zeta }{|}_{\zeta =\pm b}=0. \end{array}$$

The solution of (3.21) is given by

3.22 $$\Psi =\frac{32\nu ab^{2}}{\pi^{3}}\sum_{n=1}^{\infty }C_{n}\sin\left(\frac{(2n-1)\pi }{2b}\zeta \right)\cosh\left(\frac{(2n-1)\pi }{2b}\eta \right),$$

within which

3.23 $$C_{n}=\frac{{\left(-1\right)}^{n}}{{\left(2n-1\right)}^{3}}\text{csch}\left(\frac{(2n-1)\pi a}{2b}\right).$$

As a result, we have

3.24 $$\sigma_{12}^{\left(0\right)}=\frac{{\tilde{w}}_{0}^{\prime \prime \prime }}{2(1+\nu )}[\Psi_{,\eta }+2\nu \eta \zeta ]\;\mathrm{and}\;\sigma_{13}^{\left(0\right)}=\frac{{\tilde{w}}_{0}^{\prime \prime \prime }}{2(1+\nu )}[\Psi_{,\zeta }+\zeta^{2}-b^{2}].$$

It can be readily verified that substitution of (3.24)_2_ into (3.5) and integration over the cross section *R* once again gives us equation (3.16) that governs ${\tilde{w}}_{0}$. Therefore, we have now satisfied all of the *O*(*ε*^2^) equations (3.3)–(3.11). To obtain a non-trivial correction term(s) for the governing equation (3.16), we now have to proceed to the next asymptotic order.

## 4. The higher-order theory

The consideration of *O*(*ε*^4^) terms after substituting asymptotic ansatz (2.8) into equations (2.5) and (2.6) results in the following system of governing equations:

4.1 $$\begin{array}{rl} & \sigma_{11,\xi }^{\left(2\right)}+\sigma_{12,\eta }^{\left(2\right)}+\sigma_{13,\zeta }^{\left(2\right)}=u_{,\tau \tau }^{\left(0\right)}, \end{array}$$

4.2 $$\begin{array}{rl} & \sigma_{31,\xi }^{\left(2\right)}+\sigma_{32,\eta }^{\left(2\right)}+\sigma_{33,\zeta }^{\left(2\right)}=w_{,\tau \tau }^{\left(2\right)} \end{array}$$

and

4.3 $$\begin{array}{rl} (1+\nu )(1-2\nu )\sigma_{11}^{\left(2\right)} & \;=(1-\nu )u_{,\xi }^{\left(2\right)}+\nu v_{,\eta }^{\left(2\right)}+\nu w_{,\zeta }^{\left(4\right)}, \end{array}$$

4.4 $$\begin{array}{rl} (1+\nu )(1-2\nu )\sigma_{22}^{\left(0\right)} & \;=\nu u_{,\xi }^{\left(2\right)}+(1-\nu )v_{,\eta }^{\left(2\right)}+\nu w_{,\zeta }^{\left(4\right)}, \end{array}$$

4.5 $$\begin{array}{rl} (1+\nu )(1-2\nu )\sigma_{33}^{\left(0\right)} & \;=\nu u_{,\xi }^{\left(2\right)}+\nu v_{,\eta }^{\left(2\right)}+(1-\nu )w_{,\zeta }^{\left(4\right)} \end{array}$$

(note that we omitted here the equations that will not be required for deriving the refined governing equation for $\tilde{w}$).

We begin solving system (4.1)–(4.5) by integrating equation (4.2) over the cross section *R*. If conditions on free faces are taken into account, we obtain

4.6 $$\iint_{R}\sigma_{13,\xi }^{\left(2\right)}\;d\eta \;d\zeta =\iint_{R}w_{,\tau \tau }^{\left(2\right)}\;d\eta \;d\zeta .$$

As the next step, we multiply equation (4.1) by *ζ* and integrate over the cross section to determine that

4.7 $$\iint_{R}\sigma_{13}^{\left(2\right)}\;d\eta \;d\zeta =\iint_{R}\zeta (\sigma_{11,\xi }^{\left(2\right)}-u_{,\tau \tau }^{\left(0\right)})\;d\eta \;d\zeta .$$

The stress component $\sigma_{11}^{\left(2\right)}$ on the right-hand side can be expressed using equations (4.3)–(4.5) in the form

4.8 $$\sigma_{11}^{\left(2\right)}=\nu (\sigma_{22}^{\left(0\right)}+\sigma_{33}^{\left(0\right)})+u_{,\xi }^{\left(2\right)},$$

and substituted back into the right-hand side of (4.7). In addition, by multiplying equations (3.4), (3.5) and (3.5) by *ηζ*, $\frac{1}{2}\eta^{2}$ and $\frac{1}{2}\zeta^{2}$, respectively, and integrating the obtained equations over the cross-section, we obtain the following identities:

4.9 $$\begin{array}{rl} \iint_{R}\zeta \sigma_{22}^{\left(0\right)}\;d\eta \;d\zeta & \;=\iint_{R}\eta \zeta \sigma_{12,\xi }^{\left(0\right)}\;d\eta \;d\zeta -\iint_{R}\eta \sigma_{23}^{\left(0\right)}\;d\eta \;d\zeta , \end{array}$$

4.10 $$\begin{array}{rl} \iint_{R}\eta \sigma_{23}^{\left(0\right)}\;d\eta \;d\zeta & \;=-\iint_{R}\frac{1}{2}\eta^{2}({\tilde{w}}_{0,\tau \tau }-\sigma_{13,\xi }^{\left(0\right)})\;d\eta \;d\zeta \end{array}$$

4.11 $$\begin{array}{rl} \text{and}\;\iint_{R}\zeta \sigma_{33}^{\left(0\right)}\;d\eta \;d\zeta & \;=-\iint_{R}\frac{1}{2}\zeta^{2}({\tilde{w}}_{0,\tau \tau }-\sigma_{13,\xi }^{\left(0\right)})\;d\eta \;d\zeta . \end{array}$$

By combining these results together, one arrives at the equality

4.12 $$\begin{array}{r} \iint_{R}\zeta \sigma_{11}^{\left(2\right)}\;d\eta \;d\zeta =\iint_{R}\left\{\nu \left[\eta \zeta \sigma_{12,\xi }^{\left(0\right)}+\frac{1}{2}(\eta^{2}-\zeta^{2})({\tilde{w}}_{0,\tau \tau }-\sigma_{13,\xi }^{\left(0\right)})\right]+\zeta u_{,\xi }^{\left(2\right)}\right\}d\eta \;d\zeta . \end{array}$$

In view of the specific structure of expressions for $\sigma_{ij}^{\left(k\right)}$, see (2.8) and (2.9), we differentiate equation (4.12) with respect to *ξ* and substitute the result into the right-hand side of equation (4.7). Then we differentiate equation (4.7) with respect to *ξ* and substitute the result into the left-hand side of (4.6). The resulting expression for (4.6) is given by

4.13 $$\iint_{R}\left\{\frac{\partial^{6}{\tilde{w}}_{0}}{\partial \xi^{6}}\Phi (\eta ,\zeta )+\frac{\partial^{4}{\tilde{w}}_{0}}{\partial \xi^{2}\partial \tau^{2}}[\nu \eta^{2}+(1-\nu )\zeta^{2}]-\frac{\partial^{4}{\tilde{w}}_{2}}{\partial \xi^{4}}\zeta^{2}-\frac{\partial^{2}{\tilde{w}}_{2}}{\partial \tau^{2}}\right\}d\eta \;d\zeta =0,$$

in which

4.14 $$\begin{array}{rl} \Phi (\eta ,\zeta ) & \;=\zeta \Psi +\frac{\nu }{4(1+\nu )}\{2\eta \zeta \frac{\partial \Psi }{\partial \eta }+(\zeta^{2}-\eta^{2})\frac{\partial \Psi }{\partial \zeta }\\ & \;+(6\nu +1)\eta^{2}\zeta^{2}+\frac{\left(4+5\nu -2\nu^{2}\right)}{3\nu }\zeta^{4}-\frac{\left(4+5\nu \right)}{\nu }b^{2}\zeta^{2}+b^{2}\eta^{2}\}. \end{array}$$

The integral within equation (4.13) can be computed explicitly, yielding the governing equation for ${\tilde{w}}_{2}$ in the form

4.15 $$\frac{b^{2}}{3}\frac{\partial^{4}{\tilde{w}}_{2}}{\partial \xi^{4}}+\frac{\partial^{2}{\tilde{w}}_{2}}{\partial \tau^{2}}+k_{22}\frac{\partial^{4}{\tilde{w}}_{0}}{\partial \xi^{2}\partial \tau^{2}}+k_{60}\frac{\partial^{6}{\tilde{w}}_{0}}{\partial \xi^{6}}=0,$$

with the coefficients

4.16 $$\begin{array}{rl} k_{22} & \;=-\frac{b^{2}}{3}[1-\nu +\nu q^{2}]\\ \text{and}\;\;k_{60} & \;=\frac{b^{4}}{45(1+\nu )}[12+27\nu +3\nu^{2}-5q^{2}\nu (1+3\nu )+15\nu^{2}qS(q)], \end{array}$$

defined using

4.17 $$q=\frac{a}{b}\equiv \frac{h_{2}}{h_{3}}\;\text{and}\;S(q)=\frac{384}{\pi^{5}}\sum_{n=1}^{\infty }\frac{\coth(q\pi (2n-1)/2)}{{\left(2n-1\right)}^{5}}.$$

Keeping in mind the form of the original asymptotic expansion (2.8) and, specifically, the fact that $\tilde{w}={\tilde{w}}_{0}+\varepsilon^{2}{\tilde{w}}_{2}+O(\varepsilon^{4})$, we can combine equations (3.16) and (4.15) into

4.18 $$\frac{b^{2}}{3}\frac{\partial^{4}\tilde{w}}{\partial \xi^{4}}+\frac{\partial^{2}\tilde{w}}{\partial \tau^{2}}+\varepsilon^{2}\left[k_{22}\frac{\partial^{4}\tilde{w}}{\partial \xi^{2}\partial \tau^{2}}+k_{60}\frac{\partial^{6}\tilde{w}}{\partial \xi^{6}}\right]=0,$$

which can be re-written in an asymptotically equivalent form

4.19 $$\frac{b^{2}}{3}\frac{\partial^{4}\tilde{w}}{\partial \xi^{4}}+\frac{\partial^{2}\tilde{w}}{\partial \tau^{2}}-\varepsilon^{2}k_{0}(\nu ,q)\frac{b^{2}}{3}\frac{\partial^{4}\tilde{w}}{\partial \xi^{2}\partial \tau^{2}}=0,$$

where

4.20 $$k_{0}(\nu ,q)=\frac{1}{5(1+\nu )}[17+27\nu -2\nu^{2}-10\nu^{2}q^{2}+15\nu^{2}qS(q)].$$

Equation (4.19) can also be re-scaled back to the dimensional form, yielding

4.21 $$\frac{Eh_{3}^{2}}{3\rho }\frac{\partial^{4}u_{3}}{\partial x_{1}^{4}}+\frac{\partial^{2}u_{3}}{\partial t^{2}}-\frac{1}{3}h_{3}^{2}k_{0}(\nu ,q)\frac{\partial^{4}u_{3}}{\partial x_{1}^{2}\partial t^{2}}=0.$$

This equation, together with the expression for a non-dimensional coefficient *k*_0_(*ν*,*q*), constitute the main result of this paper. We will now turn to the assessment of the qualitative and quantitative behaviour of the newly derived beam equation.

## 5. Numerical examples

Before we discuss the numerical performance of the beam equation (4.21), it is worth making few remarks about practical computation of the function *S*(*q*) used to define *k*_0_(*ν*,*q*), see (4.17)_2_. First, given a fixed *q*, the infinite series within (4.17)_2_ is absolutely convergent and converges very rapidly. The first two terms of the series already provide relative errors below 0.1%; the first three terms result in relative errors below 0.01%. An asymptotic expansion for *q*≪1 has the form

5.1 $$S(q)=\frac{4}{5q}+\frac{2}{3}q-\frac{2}{15}q^{3}+\cdots .$$

A useful representation of (4.17)_2_ for *q*≫1 can be formulated by using the fact that $\coth x=1+2/({\text{e}}^{2x}-1)$; the results outlined in appendix A, especially equation (A.4), indicate that

5.2 $$S(q)=\frac{372}{\pi^{5}}\zeta (5)+\frac{768}{\pi^{5}}\sum_{n=1}^{\infty }\frac{1}{{\left(2n-1\right)}^{5}}\frac{1}{e^{\pi q(2n-1)}-1},$$

where *ζ*(*x*) is the Riemann zeta function and *ζ*(5)≈1.0369278.

We can now turn our attention to assessing how well the new equation reproduces the dynamics of rectangular beams. This can be done by comparing the dispersion curves of the beam modelled by the full three-dimensional theory with the dispersion curves predicted by the one-dimensional model. The dispersion is characterized by looking at plane wave solutions of the form

5.3 $$u_{i}(\text{x},t)=U_{i}(x_{2},x_{3})\exp(\;ik(x_{1}-vt)),\;i=1,2,3,$$

where *k* is the wavenumber and *v* the phase velocity. A closed-form analytical expression for the dispersion relation describing *v*=*v*(*k*) is not available, so the dispersion is usually studied using numerical methods (e.g. [26,27]). We computed numerical solutions of the relevant dispersion relation using the semi-analytical finite-element (SAFE) method, which is a technique that involves the analytic separation of the axial variable followed by the finite-element solution of the suitably parametrized cross-sectional problem (e.g. [28–30] and references therein). All finite-element computations in this paper were performed using the COMSOL Multiphysics 5.1.

**Figure 2.**
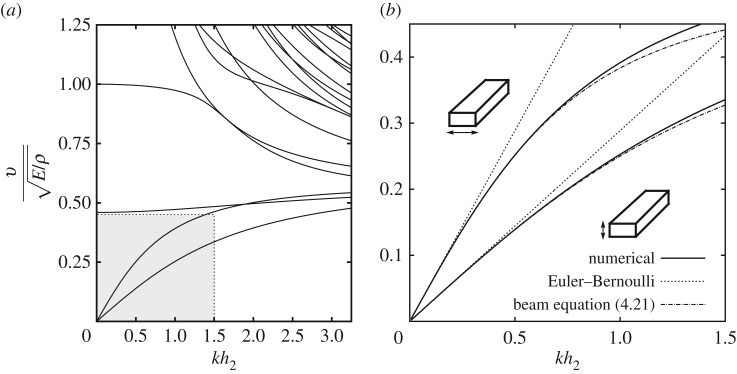
Non-dimensional phase velocity $v/\sqrt{E/\rho }$ as the function of non-dimensional wave number *kh*_2_ for a rectangular beam 2×1 with *ν*=0.3. For the lower bending mode *q*=2, for the upper bending mode *q*=1/2. (*a*) The dispersion curves obtained using the SAFE method. The shaded area, also shown zoomed as (*b*), presents a comparison between two bending modes (solid lines) and their approximations using the Euler–Bernoulli theory and asymptotic equation (4.21) (dotted and dash-dotted lines, respectively).

The first numerical example that we considered concerns a rectangular beam with cross section 2×1. The dispersion curves for the beam are shown in figure 2*a*. Our particular interest is in the long-wave behaviour of two lowest (bending) modes. Both of the relevant curves are shown in the shaded area of the graph and, also, presented magnified in figure 2*b*. Finite-element solutions (the solid curves) are shown together with predictions of the Euler–Bernoulli beam theory (dotted lines), as well as predictions of the new asymptotic equation (4.21) (dash-dotted curves). More specifically, the relevant dispersion relations can be obtained by inserting (5.3) into equation (4.21) and re-arranging the result as

5.4 $$\frac{v^{2}}{E/\rho }=\frac{I_{x_{2}}k^{2}/A}{1+k_{0}(\nu ,q)I_{x_{2}}k^{2}/A}=\frac{I_{x_{2}}k^{2}}{A}\left(1-k_{0}(\nu ,q)\frac{I_{x_{2}}k^{2}}{A}+O\left(\frac{I_{x_{2}}^{2}k^{4}}{A^{2}}\right)\right),$$

where *I*_*x*_2__=2*h*_2_(2*h*_3_)^3^/12 and *A*=4*h*_2_*h*_3_, just like we mentioned in §3. The dispersion relation for the Euler–Bernoulli theory is obtained when the *O*(*I*_*x*_2__*k*^2^/*A*) term is omitted on the right-hand side; the dispersion relation for the new beam equation must include this term. Equation (5.4) approximates a single (lower) dispersion curve that corresponds to bending in the plane *Ox*_1_*x*_3_. The bending in the plane *Ox*_1_*x*_2_ can be described by replacing *I*_*x*_2__ and *q* in equation (5.4) by *I*_*x*_3__=(2*h*_2_)^3^2*h*_3_/12 and 1/*q*, respectively, see the upper dispersion curve in figure 2*b*. The improved accuracy of the new equation compared to the accuracy of the classical Euler–Bernoulli equation is evident; the classical theory overestimates the phase velocity, just as expected.

Equation (5.4) predicts no dispersion when *k*_0_(*ν*,*q*)=0; of course, this actually means that dispersion in this case would be governed by the next $O(I_{x_{2}}^{2}k^{4}/A^{2})$ term. For example, in a rectangular beam with the cross section 6.2916×1 made of a steel with *ν*=0.3, the correction term in equation (4.21) vanishes, so one expects to see a substantial improvement in the performance of the Euler–Bernoulli theory for the lower bending mode. For even larger aspect ratios, *k*_0_(*ν*,*q*) becomes negative; it does not quite mean the change of the type of dispersion, that e.g. happens for shallow water waves, but it does mean that velocities predicted by the Euler–Bernoulli theory may actually underestimate the true phase velocity of the lower bending mode at low frequencies. This is contrary to the common intuition suggesting that the Euler–Bernoulli theory overestimates the phase velocity in a beam.

**Figure 3.**
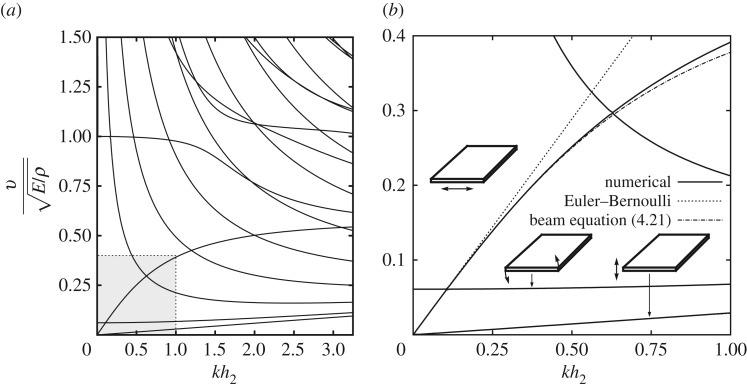
Non-dimensional phase velocity $v/\sqrt{E/\rho }$ as the function of non-dimensional wave number *kh*_2_ for a rectangular beam 20×1 with *ν*=0.3. For the lower bending mode *q*=20, for the upper bending mode *q*=1/20. (*a*) The dispersion curves obtained using the SAFE method. The shaded area, also shown zoomed as (*b*), presents a comparison between two bending modes (solid lines) and their approximations using the Euler–Bernoulli theory and asymptotic equation (4.21) (dotted and dash-dotted lines, respectively). A twisting mode is also shown.

**Figure 4.**
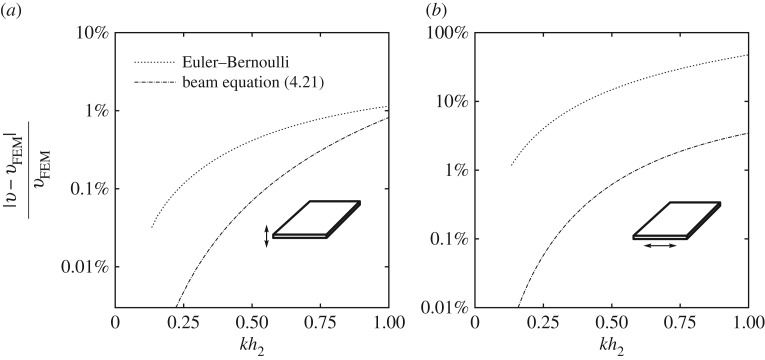
Relative approximation errors for long-wave low-frequency phase velocity of the bending modes in a rectangular beam 20×1 with *ν*=0.3. (*a*) The lower frequency (plate strip) bending mode. (*b*) The higher frequency in-plane (plane stress) bending mode.

In order to illustrate this situation, we also computed dispersion curves for the somewhat extreme case of a beam with cross section 20×1 (figure 3*a*). Similar to the previous example, we focused on the long-wave low-frequency modes, i.e. modes within the shaded area of the graph that is also presented magnified in figure 3*b*. The improved approximation accuracy for the upper bending mode, for which *q*=1/20, is easy to assess from the figure. Phase velocity of the lower bending mode, for which *q*=20, is so low that all three curves—the numerical dispersion curve, as well as its Euler–Bernoulli approximation and prediction by equation (4.21)—overlap and appear indistinguishable. This is why a separate figure 4 presents numerically computed relative approximation errors for both (*a*) lower and (*b*) upper bending modes. The relative error of the Euler–Bernoulli theory for lower mode barely exceeds 1%, which explains our observations (figure 4*a*). The relative error of the Euler–Bernoulli theory for the higher bending mode is significantly larger (figure 4*b*). In both cases, the relative errors of the new bending equation (4.21) are one to two orders of magnitude smaller than relative errors of the classical theory.

As we assumed the Poisson ratio *ν*=0.3 and since for lower bending mode *q*=20, the correction coefficient *k*_0_(0.3,20)≈−46.315<0. Therefore, just as we indicated earlier, for lower bending mode in flat plate-like beams the Euler–Bernoulli theory tends to underestimate the phase velocity (this is related to, but is not the same thing as the negative values for Timishenko's shear correction factor, which will be discussed in §7). Of course, proper modelling of bending waves in such a structure is likely to also require properly accounting for twisting motions of a beam (figure 3*b*). Therefore, in situations featuring low-frequency propagation/interaction of several bending modes of flat beams, one would probably benefit more from using a plate theory instead of a single mode beam equation such as (4.21).

## 6. The limiting case of thin flat beams

**Figure 5.**
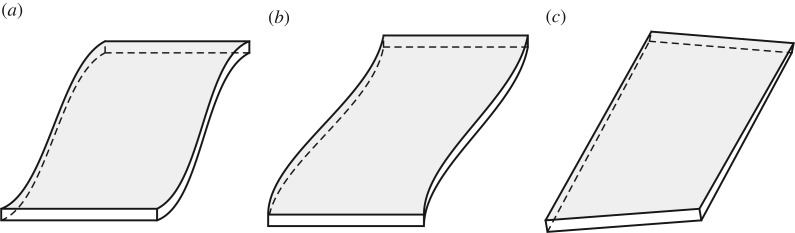
Three modes that dominate a long-wave response of a flat beam: (*a*) a lower (out-of-plane) bending mode, (*b*) a higher (in-plane) bending mode and (*c*) a twisting mode.

Let us designate thin, flat flexural elements of rectangular cross section and small (or large) width to thickness aspect ratio as simply thin flat beams. Such beams, e.g. the beam with aspect ratio 20×1 that we studied in §5, are special in that they can be meaningfully modelled using thin plate approximations. It can be gathered from figure 3*b* that long-wave response of a thin flat beam is dominated by three modes: two bending modes (in-plane and out-of-plane), as well as a twisting mode. Typical beam deformations that correspond to these modes are schematically illustrated in figure 5. The twisting mode shown in figure 5*c* is distinct from the bending modes in that it is not a low-frequency mode; its long-wave limit corresponds to a relatively low, yet non-zero frequency. This frequency would be a natural limit for the applicability of beam equation (4.21) for thin flat beams. We are not going to otherwise study the twisting mode and leave its analysis to a separate publication.

The lower bending mode (figure 5*a*) corresponds to the asymptotic limit when *q*≡*h*_2_/*h*_3_≫1. The infinite sum on the right-hand side of equation (5.2) is exponentially small in this case; hence, $S(\infty )=372\zeta (5)/\pi^{5}\approx 1.2604978$ and

6.1 $$k_{0}(\nu ,q)=-\frac{\nu^{2}}{1+\nu }(2q^{2}-3qS(\infty ))+O(1),\;q\gg 1.$$

This expansion shows that for all sufficiently flat beams the Euler–Bernoulli theory will underestimate the true phase velocity of plane waves, just as we observed previously for the rectangular beam 20×1.

The correctness of expansion (6.1) can be confirmed using a classical Kirchhoff plate theory. A thin flat beam can be thought of as a plate strip of width 2*h*_2_ and thickness 2*h*_3_; beam bending mode would correspond to the fundamental symmetric mode of the strip. Motion of the plate strip of thickness 2*h*_3_ is governed by the equation

6.2 $$\frac{2Eh_{3}^{3}}{3(1-\nu^{2})}\left(\frac{\partial^{4}u_{3}}{\partial x_{1}^{4}}+2\frac{\partial^{4}u_{3}}{\partial x_{1}^{2}\partial x_{2}^{2}}+\frac{\partial^{4}u_{3}}{\partial x_{2}^{4}}\right)+2\rho h_{3}\frac{\partial^{2}u_{3}}{\partial t^{2}}=0.$$

The boundary conditions that ensure that strip edges are stress-free can be written in the form

6.3 $$\frac{\partial^{2}u_{3}}{\partial x_{2}^{2}}+\nu \frac{\partial^{2}u_{3}}{\partial x_{1}^{2}}+Kh_{3}(1-\nu )\frac{\partial^{3}u_{3}}{\partial x_{1}^{2}\partial x_{2}}=0$$

and

6.4 $$\frac{\partial^{3}u_{3}}{\partial x_{2}^{3}}-(2-\nu )\frac{\partial^{3}u_{3}}{\partial x_{1}^{2}\partial x_{2}}=0,$$

where *x*_2_=±*h*_2_. These conditions are unusual due to the presence of *O*(*h*_3_) term in (6.3), which represents a first non-trivial correction to the classical Kirchhoff boundary conditions by Kolos [18]. Without this correction term, the classical leading-order theory of plate bending would only be able to capture the leading-order behaviour of expansion (6.1); with the correction term added we expect to recover (6.1) fully. Indeed, Kolos [18, eqn (2.13)] showed that the constant *K* is defined as

6.5 $$K=\frac{384}{\pi^{5}}\sum_{n=1}^{\infty }\frac{1}{{\left(2n-1\right)}^{5}}.$$

That is, in our notation K≡S(∞), which immediately suggests the potential relevance of the correction term.

Solutions of the form

6.6 $$u_{3}=(U_{3}^{\left(1\right)}\cosh(\alpha_{1}kh_{2})+U_{3}^{\left(2\right)}\cosh(\alpha_{2}kh_{2}))\exp(\;ik(x_{1}-vt)),$$

satisfy governing equation (6.2) as long as

6.7 $$\alpha_{1,2}^{2}=1\pm \frac{\sqrt{3(1-\nu^{2})}}{kh_{3}}\frac{v}{\sqrt{E/\rho }}.$$

Substitution into boundary conditions (6.3) and (6.4) shows that non-trivial solutions of form (6.6) only exist when the following secular equation is satisfied:

6.8 $$g(\alpha_{1},\alpha_{2},\nu )C_{1}S_{2}-g(\alpha_{2},\alpha_{1},\nu )S_{1}C_{2}+kh_{3}K(1-\nu )\alpha_{1}\alpha_{2}(\alpha_{1}^{2}-\alpha_{2}^{2})S_{1}S_{2}=0,$$

within which

6.9 $$S_{i}=\sinh(\alpha_{i}kh_{2}),\;C_{i}=\cosh(\alpha_{i}kh_{2})$$

and

6.10 $$g(\alpha_{i},\alpha_{j},\nu )=\alpha_{j}(\alpha_{i}^{2}-\nu )(\alpha_{j}^{2}+\nu -2),\;i\neq j,\;i,j=1,2.$$

If *K* was equal to zero, equation (6.8) would reduce to the dispersion relation first obtained and analysed by Konenkov [31]. As is, this secular equation represents a generalization of Konenkov's result to the case of non-vanishing ratios *h*_3_/*h*_2_.

Although (6.8) cannot, in general, be solved analytically, formal asymptotic expansions are possible and expansion in the long-wave low-frequency limit has the following form:

6.11 $$\frac{v^{2}}{E/\rho }=\frac{I_{x_{2}}k^{2}}{A}\left(1+\frac{\nu^{2}(2q^{2}-3Kq)}{1+\nu }\frac{I_{x_{2}}k^{2}}{A}+O\left(\frac{I_{x_{2}}^{2}k^{4}}{A^{2}}\right)\right),$$

which in view of equations (5.4) and (6.5) is clearly equivalent to (6.1). This serves to confirm the validity of (5.4), at least in the asymptotic sense for *q*≫1, and, simultaneously, this serves as a non-trivial confirmation of the correctness of the boundary layer adjustment term by Kolos [18] for the free boundary conditions in the Kirchhoff theory.

The higher bending mode, sketched in figure 5*b*, is associated with the values of parameter *q*≡*h*_2_/*h*_3_≪1. A mode like this can also be extrapolated by using a plate theory; however, the bending deformation in this case would be confined to a plane and, therefore, would require an application of the plane stress theory. Timoshenko [19] and Stephen [11] considered this limiting behaviour and obtained, in different notations, the following expansion for the phase velocity:

6.12 $$\frac{v^{2}}{E/\rho }=\frac{I_{x_{2}}k^{2}}{A}\left(1-\left(2\nu +\frac{17}{5}\right)\frac{I_{x_{2}}k^{2}}{A}+O\left(\frac{I_{x_{2}}^{2}k^{4}}{A^{2}}\right)\right).$$

For our new beam bending equation, this corresponds to the limit when *q*≡*h*_2_/*h*_3_≪1; hence, in view of expansion (5.1), we can say that

6.13 $$k_{0}(\nu ,q)=2\nu +\frac{17}{5}-\frac{2\nu^{2}}{5(1+\nu )}q^{4}+\cdots ,\;q\ll 1.$$

A quick glance at dispersion relation (5.4) now confirms that Stephen's expansion (6.12) gives the correct leading-order behaviour for the dispersion coefficient *k*_0_(*ν*,*q*) when *q*≪1.

It would be interesting to explore whether a refined plane stress theory would be sufficient to also recover the *O*(*q*^4^) correction term in (6.13). The relevant higher-order equations are available in [4] and the appropriately refined boundary conditions are described in [18]. However, this discussion would take us too far outside of the scope of this paper.

## 7. Shear correction factor

In derivation of his beam equation, Timoshenko relied upon two key constitutive assumptions:

7.1 $$M=-EI_{x_{2}}\frac{\partial \phi }{\partial x_{1}}\;\text{and}\;Q=\kappa \mu A\left(\frac{\partial w}{\partial x_{1}}-\phi \right),$$

where *M* is the bending moment, *ϕ* the angle characterizing rotation of a cross-sectional element, *Q* the shearing force, *A* the cross-sectional area and *μ*=*E*/2(1+*ν*) the shear modulus, see [8]. Assumption (7.1)_1_ encapsulates the same leading-order relationship between bending moment *M* and the radius of curvature (∂*ϕ*/∂*x*_1_)^−1^ as in the Euler–Bernoulli theory. Assumption (7.1)_2_ that relates shearing force at the cross section to transverse shear strain at the centroidal axis is unique to Timoshenko's theory. The proportionality constant *κ*—‘the shear correction factor’—had to be formally introduced to bring into accord the relationship that was known to Timoshenko to be approximate at best. The associated beam equation, unsurprisingly, also depends on *κ*:

7.2 $$EI_{x_{2}}\frac{\partial^{4}w}{\partial x_{1}^{4}}+\rho A\frac{\partial^{2}w}{\partial t^{2}}-\rho I_{x_{2}}\left(1+\frac{E}{\kappa \mu }\right)\frac{\partial^{4}w}{\partial x_{1}^{2}\partial t^{2}}+\frac{\rho^{2}I_{x_{2}}}{\kappa \mu }\frac{\partial^{4}w}{\partial t^{4}}=0,$$

(see [9]). It is important to stress that, even though equation (7.2) contains higher-order terms compared to the Euler–Bernoulli equation, the associated higher-order corrections are not necessarily consistent with the appropriately truncated expressions from the three-dimensional theory. This happens because the underlying assumptions (7.1) are not accurate to the same order of truncation, see [9] and also discussions of similar issues in refined theories of plates and shells in [3,32]. More specifically, even if the constitutive relation for shearing force (7.1)_2_ is made asymptotically consistent, the recovery of the appropriately refined displacement and stress fields would also require a higher-order generalization of the constitutive relation for bending moment (7.1)_1_ as well as appropriate refinements to the transverse inertia. This can be deduced from asymptotic formulae in § 4, see also [3,32] and references therein for more details. As a result, even with the presence of ‘tuning’ parameter *κ*, one does not expect to be able to recover correct asymptotic behaviour of every aspect of the resulting theory. In fact, later in this section, we will demonstrate how the choice of *κ* that increases only the numerical accuracy of equation (7.1)_2_ is resulting in inconsistent governing equation (7.2).

A sizeable body of the literature sprung up from attempts to either formulate a consistent method to derive *κ* or to justify a particular expression for it. Timoshenko himself used several cross-section-independent constant values [8,19]; a surprisingly influential paper by Cowper [20] used a series of approximations to arrive at a general expression for *κ* that was then specialized to a number of canonical cross sections; for rectangular beams, it gives

7.3 $$\kappa_{C}=\frac{10(1+\nu )}{12+11\nu }.$$

Stephen [11] used several long-wave low-frequency expansions (including (6.12)) to show that Cowper's expressions cannot be consistent, at least for circular and rectangular cross sections.

The same idea can also be applied to our beam equation (4.21) to derive an asymptotically consistent expression for the Timoshenko shear correction factor. The appropriate dispersion relation can be obtained by substituting plane wave solution (5.3) into (7.2) and expanding the result for small values of *I*_*x*_2__*k*^2^/*A*. This procedure yields the following approximate dispersion relation:

7.4 $$\frac{v^{2}}{E/\rho }=\frac{I_{x_{2}}k^{2}}{A}\left(1-\left(1+\frac{E}{\kappa \mu }\right)\frac{I_{x_{2}}k^{2}}{A}+O\left(\frac{I_{x_{2}}^{2}k^{4}}{A^{2}}\right)\right).$$

A direct comparison of expansions (7.4) and (5.4) indicates that Timoshenko's equation will be asymptotically equivalent to beam equation (4.21) as long as

7.5 $$\begin{array}{rl} \kappa_{\mathrm{NPK}} & \;=\frac{2(1+\nu )}{k_{0}(\nu ,q)-1}=\frac{5{\left(1+\nu \right)}^{2}}{6+11\nu -\nu^{2}-5\nu^{2}q^{2}+\frac{2880\nu^{2}q}{\pi^{5}}\sum_{n=1}^{\infty }\frac{\coth(q\pi \frac{2n-1}{2})}{{\left(2n-1\right)}^{5}}}, \end{array}$$

which appears to be a new expression for the Timoshenko shear correction factor. This expression can be compared with other expressions for *κ* in the literature. Stephen & Levinson [21, eqn (54b)] published an *ad hoc* derivation that led to the following expression:

7.6 $$\kappa_{\mathrm{SL}}=\frac{5{\left(1+\nu \right)}^{2}}{6+11\nu +\nu^{2}\left(5-q^{4}+\frac{90q^{5}}{\pi^{5}}\sum_{n=1}^{\infty }\frac{\tanh(\pi n/q)}{n^{5}}\right)},$$

which, although appears different to (7.5), is equivalent to (7.5), see the demonstration in appendix A. The same expression for *κ* was confirmed by Stephen [22, eqn (31)] and, in a somewhat different form, by Hutchinson [23, eqn (47)], see the discussion in [24]. In addition, a direct perturbation in powers of thickness variable was developed by Chan *et al.* [25], which also confirmed the correctness of (7.6). Based on these comments, we conclude that *κ*_NPK_=*κ*_SL_ and that equations (7.5) and (7.6) are equivalent and asymptotically correct forms for the shear correction factor for a rectangular Timoshenko beam.

The infinite series used in equations (7.5) and (7.6) will have to be truncated for numerical computations. If these series are truncated after the same number of terms, equation (7.5) provides a more precise answer for q≳1 and equation (7.6) provides a more precise answer for q≲1. However, the precision of (7.6) with truncated series quickly deteriorates for larger values of *q*, whereas the precision of (7.5) with truncated series stays approximately uniform for all values of *q* (e.g. the first 10 terms in the series are typically sufficient to obtain five significant digits of *κ*). Thus, equation (7.5) can be recommended as more universal expression for computing *κ*.

Berdichevskii & Kvashnina made a similar comparison of their asymptotic beam theory [12, eqn (3.2)] with the Timoshenko equation. This led them to derive a general expression for *κ* valid for sufficiently symmetric cross sections, see [12, eqn (8.6)]. Their resulting coefficient for circular cross sections [12, eqn (8.7)] matches the coefficient derived from three-dimensional theory by Stephen [11] and since then confirmed by a number of other researchers. At the same time, their coefficient for rectangular cross sections [12, eqn (8.9)]:

7.7 $$\begin{array}{r} \kappa_{\mathrm{BK}}=\frac{1}{\frac{191\nu^{2}+594\nu +324}{270{\left(1+\nu \right)}^{2}}+\frac{\nu^{2}}{{\left(1+\nu \right)}^{2}}\left(\frac{5}{54q^{4}}+\frac{6q}{\pi^{5}}\sum_{n=1}^{\infty }\frac{\tanh(\pi n/q)}{n^{5}}\right)}, \end{array}$$

despite certain similarities, is not equivalent to (7.5) or (7.6) and, therefore, appears to be in error.

Substantial research effort has been spent trying to evaluate the value of *κ* that would render approximate formula (7.1)_2_ numerically exact for rectangular beams. The earliest relevant result in the literature was obtained by Eliseev [33, eqn (33)]; it can be re-written in our notation as:

7.8 $$\kappa_{E}=\frac{{\left(1+\nu \right)}^{2}}{\frac{6}{5}(1+2\nu +2\nu^{2})-\frac{576\nu^{2}q}{\pi^{5}}\sum_{n=1}^{\infty }\frac{\coth(q\pi \frac{2n-1}{2})}{{\left(2n-1\right)}^{5}}+\nu^{2}q^{2}}.$$

Fully equivalent, although very different-looking, shear correction factors were obtained by Renton [34], just above eqn (27):

7.9 $$\kappa_{R}=\frac{1}{\frac{6}{5}+{\left(\frac{\nu }{1+\nu }\right)}^{2}\sum_{m=0}^{\infty }\sum_{n=1}^{\infty }\frac{36q^{4}}{\pi^{6}{\left(2m+1\right)}^{2}n^{2}(4n^{2}+{\left(2m+1\right)}^{2}q^{2})}},$$

and by Yu & Hodges [14, eqn (68)_1_]:

7.10 $$\kappa_{\mathrm{YH}}=\frac{1}{\frac{6}{5}+q^{4}{\left(\frac{\nu }{1+\nu }\right)}^{2}\left(\frac{1}{5}-\frac{18q}{\pi^{5}}\sum_{n=1}^{\infty }\frac{\tanh(\pi n/q)}{n^{5}}\right)}.$$

The equivalence of expressions (7.8)–(7.10) can be easily demonstrated using the identities described in appendix A, so *κ*_E_=*κ*_R_=*κ*_YH_.

**Figure 6.**
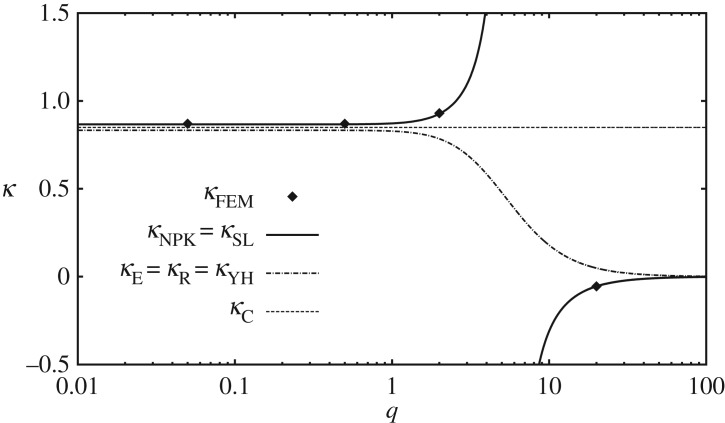
The shear correction factor *κ* for rectangular beams when the Poisson ratio *ν*=0.3. Parameter *q* is the aspect ratio of the cross section rectangle.

Despite some superficial similarities between the expressions for *κ*_NPK_=*κ*_SL_ and expressions for *κ*_E_=*κ*_R_=*κ*_YH_, it is important to stress that they are very different functions of the beam aspect ratio *q* and the Poisson ratio *ν*. Figure 6 illustrates this point for the Poisson ratio *ν*=0.3. It is immediately obvious that expressions *κ*_E_=*κ*_R_=*κ*_YH_ do not result in the correct limiting behaviour for *q*≪1, just like *κ*_C_ did not. The definition of *κ* that leads to the asymptotically consistent governing equation is actually discontinuous when *k*_0_(*ν*,*q*)=1, see (7.5); when *ν*=0.3 this happens for *q*≈5.5671. The asymptotically correct value of *κ* for even flatter beams is negative, which suggests anti-spring relationship between shearing force and transverse shear strain. The physical meaning of equation (7.1)_2_ for negative or discontinuous values of *κ* is dubious; however, rather than casting the doubt on definitions of *κ*_NPK_=*κ*_SL_, this simply reflects the fact that (7.1)_2_ is not a consistent statement from the point of view of long-wave low-frequency asymptotics.

In fact, it is possible to estimate the ‘true’ value of *κ* from the dispersion curves obtained using the finite-element method. Indeed, we expect that Timoshenko's dispersion relation (7.4) must provide a fair approximation of the exact dispersion curves for sufficiently small wavenumbers. Therefore, the difference between the phase velocity of Euler–Bernoulli beam $v_{\mathrm{EB}}=\sqrt{E/\rho }I_{x_{2}}k^{2}/A$ and the phase velocity resulting from the full three-dimensional theory *v*_FEM_ must be equal, to the leading order, to the higher-order correction term in equation (7.4). A simple algebraic manipulation can then be used to show that

7.11 $$\kappa_{\mathrm{FEM}}\approx \frac{2(1+\nu )}{\left(2\frac{v_{\mathrm{EB}}-v_{\mathrm{FEM}}}{\sqrt{E/\rho }{\left(I_{x_{2}}k^{2}/A\right)}^{3}}-1\right)}.$$

This identity is, of course, approximate, but it becomes more and more accurate as *I*_*x*_2__*k*^2^/*A*→0. Thus, the long-wave limit of (7.11) can be used to estimate the values of *κ* that correspond to the considered dispersion curve. We applied the described procedure to all four bending mode dispersion curves discussed in §5 and obtained estimates for the shear correction factor. These estimates are denoted as *κ*_FEM_ in figure 6 and they can also be seen tabulated in table 1. Also included in table 1 are the values of the shear correction factor that are obtained from the expressions discussed in this section. From these results, it is abundantly clear that only the definitions of *κ*_NPK_=*κ*_SL_ are truly consistent with the results of finite-element simulations.

**Table 1.** Specific values of the shear correction factor for *ν*=0.3.

*q*	*κ*_C_	*κ*_E_=*κ*_R_=*κ*_YH_	*κ*_NPK_=*κ*_SL_	*κ*_FEM_
1/20	0.8497	0.8333	0.8667	0.87
1/2	0.8497	0.8329	0.8671	0.87
2	0.8497	0.7844	0.9267	0.93
20	0.8497	0.04866	−0.05495	−0.055

## 8. Summary

An asymptotic beam equation constructed in this paper can be used to describe the long-wave low-frequency behaviour of both bending modes of a rectangular beam without making any assumptions about aspect ratio of the beam. If we assume that *h*_2_≥*h*_3_ or, equivalently, that *q*≡*h*_2_/*h*_3_≥1, then the lower bending mode of the beam is governed by

8.1 $$\frac{EI_{x_{2}}}{\rho A}\frac{\partial^{4}w}{\partial x_{1}^{4}}+\frac{\partial^{2}w}{\partial t^{2}}-k_{0}(\nu ,q)\frac{I_{x_{2}}}{A}\frac{\partial^{4}w}{\partial x_{1}^{2}\partial t^{2}}=0.$$

in which *I*_*x*_2__=2*h*_2_(2*h*_3_)^3^/12, *A*=4*h*_2_*h*_3_ and the non-dimensional coefficient *k*_0_(*ν*,*q*) is defined by equations (4.20) and (4.17)_2_. The governing equation for higher bending mode can be obtained by replacing *I*_*x*_2__ and *q* with *I*_*x*_3__=(2*h*_2_)^3^2*h*_3_/12 and 1/*q*, respectively.

We used a variety of tests to demonstrate the asymptotic consistency of the newly derived equation. Specifically, we correctly recovered the asymptotic behaviour in the limit of thin flat beams modelled by a strip of Kirchhoff plate. Very remarkably, when using the classical plate theory with the Kirchhoff boundary conditions refined by Kolos [18], the first two terms of the corresponding dispersion relation were shown to fully match the dispersion relation for the new beam theory. This appears to be the first non-trivial confirmation of the correctness of Kolos’ result. We also showed that the new equation correctly reproduces the limiting case of narrow beams, which can be obtained by analysing a strip problem for a thin plate in the state of plane stress.

A comparison with the recent literature on the Timoshenko shear correction factor *κ* was also made. A new asymptotically consistent expression for *κ* was obtained in the following form:

8.2 $$\kappa =\frac{5{\left(1+\nu \right)}^{2}}{6+11\nu -\nu^{2}-5\nu^{2}q^{2}+\frac{2880\nu^{2}q}{\pi^{5}}\sum_{n=1}^{\infty }\frac{\coth(q\pi \frac{2n-1}{2})}{{\left(2n-1\right)}^{5}}}.$$

We showed that equation (8.2), although apparently new, is equivalent to the expressions for *κ* previously derived in [21–23,25], see also discussion [24]. Given the substantial differences between the methods used to obtain these expressions, our study may serve as a confirmation of the asymptotic consistency of the previously reported results.

The relative simplicity of rectangular cross section allowed us to obtain a number of explicit analytical results for rectangular beams and cross-verify predictions that resulted from considering distinct asymptotic limiting processes. The case of more general beam cross sections will necessarily be less explicit and will become the subject of a separate treatment. However, the main benefit of the asymptotically consistent beam theory, such as the one derived in this paper, is the possibility of extending our results to finite beams. Such an extension must necessarily involve an appropriate refinement of the end boundary conditions [35]. There exists a substantial body of research on refinements of edge boundary conditions for plate theories (e.g. [36–38] and references therein). We are hoping to address the sparsity of similar results for higher-order beam theories in our future work.

## Footnotes

1 A possible way to introduce *h* could be to define it as $h=\frac{1}{2}(h_{2}+h_{3})$ or, alternatively, as $h=\max(h_{2},h_{3})$. In our derivations, we implicitly assume that *h*_2_ and *h*_3_ are of the same asymptotic order; however, the resulting expansions remain valid if one of them is substantially smaller than the other.

## Data accessibility

This article has no additional data.

## Appendix A. Demonstration of equivalence of (7.5) and (7.6)

The shear correction factors (7.5) and (7.6) feature infinite sums that must be expressed in terms of each other if we are to compare them. The necessary result can be established by using the following series for hyperbolic functions [39], 1.421.2 and 1.421.4:

A 1 $$\coth\pi x=\frac{1}{\pi x}+\frac{2x}{\pi }\sum_{k=1}^{\infty }\frac{1}{x^{2}+k^{2}}$$

and

A 2 $$\tanh\frac{\pi x}{2}=\frac{4x}{\pi }\sum_{k=1}^{\infty }\frac{1}{{\left(2k-1\right)}^{2}+x^{2}}.$$

We will also need several identities related to the Riemann zeta function. First, we note that for *s*>1

A 3 $$\zeta (s)=\sum_{k=1}^{\infty }\frac{1}{k^{s}}=\sum_{k=1}^{\infty }\left[\frac{1}{{\left(2k-1\right)}^{s}}+\frac{1}{2^{s}k^{s}}\right]=\sum_{k=1}^{\infty }\frac{1}{{\left(2k-1\right)}^{s}}+\frac{\zeta (s)}{2^{s}},$$

see [39, Sect. 9.52, 9.54]. Consequently, $\sum_{k=1}^{\infty }2^{s}/{\left(2k-1\right)}^{s}=(2^{s}-1)\zeta (s)$, and since *ζ*(6)=*π*^6^/945 we can conclude that

A 4 $$\sum_{k=1}^{\infty }\frac{2^{6}}{{\left(2k-1\right)}^{6}}=\frac{\pi^{6}}{15}.$$

Similarly, it is easy to see that for *s*>1 and *r*>1

A 5 $$\sum_{n=1}^{\infty }\sum_{k=1}^{\infty }\frac{1}{n^{s}}\frac{2^{r}}{{\left(2k-1\right)}^{r}}=(2^{r}-1)\zeta (s)\zeta (r);$$

hence, because *ζ*(2)=*π*^2^/6 and *ζ*(4)=*π*^4^/90,

A 6 $$\sum_{n=1}^{\infty }\sum_{k=1}^{\infty }\frac{1}{n^{2}}\frac{2^{4}}{{\left(2k-1\right)}^{4}}=\frac{\pi^{6}}{36}\;\mathrm{and}\;\sum_{n=1}^{\infty }\sum_{k=1}^{\infty }\frac{1}{n^{4}}\frac{2^{2}}{{\left(2k-1\right)}^{2}}=\frac{\pi^{6}}{180}.$$

Identities (A.4) and (A.6) can also be computed directly using a computer algebra system such as Waterloo Maple. With all these equations in mind, one can construct the following sequence of transformations:

A 7 $$\begin{array}{rl} 2^{5}q\sum_{k=1}^{\infty }\frac{\coth(\pi (2k-1)q/2)}{{\left(2k-1\right)}^{5}} & \;\overset{\text{(}\text{A 1}\text{)}}{=}\frac{1}{\pi }\sum_{k=1}^{\infty }\frac{2^{6}}{{\left(2k-1\right)}^{6}}\left[1+2{\left(2k-1\right)}^{2}\sum_{n=1}^{\infty }\frac{1}{{\left(2k-1\right)}^{2}+{\left(2n/q\right)}^{2}}\right]\\ & \;\overset{\text{(}\text{A 4}\text{)}}{=}\frac{\pi^{5}}{15}+\frac{2^{7}}{\pi }\sum_{n=1}^{\infty }\sum_{k=1}^{\infty }\frac{1}{{\left(2k-1\right)}^{4}}\frac{1}{{\left(2k-1\right)}^{2}+{\left(2n/q\right)}^{2}}\\ & \;=\frac{\pi^{5}}{15}+\frac{2^{7}}{\pi }\sum_{n=1}^{\infty }\sum_{k=1}^{\infty }\left[\frac{{\left(2n/q\right)}^{-2}}{{\left(2k-1\right)}^{4}}-\frac{{\left(2n/q\right)}^{-4}}{{\left(2k-1\right)}^{2}}+\frac{{\left(2n/q\right)}^{-4}}{{\left(2k-1\right)}^{2}+{\left(2n/q\right)}^{2}}\right]\\ & \;\overset{\text{(}\text{A 6}\text{)}}{=}\frac{\pi^{5}}{15}+\frac{\pi^{5}q^{2}}{18}-\frac{\pi^{5}q^{4}}{90}+\frac{2^{3}q^{4}}{\pi }\sum_{n=1}^{\infty }\frac{1}{n^{4}}\sum_{k=1}^{\infty }\frac{1}{{\left(2k-1\right)}^{2}+{\left(2n/q\right)}^{2}}\\ & \;\overset{\text{(}\text{A 2}\text{)}}{=}\frac{\pi^{5}}{90}(6+5q^{2}-q^{4})+q^{5}\sum_{n=1}^{\infty }\frac{\tanh(\pi n/q)}{n^{5}}. \end{array}$$

In the view of identity (A.7), the equivalence of expressions (7.5) and (7.6) can now be established by elementary algebraic manipulations.
